# Three-Stage Cascade Information Attenuation for Opinion Dynamics in Social Networks

**DOI:** 10.3390/e26100851

**Published:** 2024-10-08

**Authors:** Haomin Wang, Youyuan Li, Jia Chen

**Affiliations:** 1 School of Management Science and Engineering, Southwestern University of Finance and Economics, Chengdu 610074, China; wanghm@swufe.edu.cn; 2 Sichuan University Humanities and Social Sciences Key Research Base—Energy Environment Carbon Neutrality Innovation Research Center, Chengdu 610059, China; 3 School of Business Administration, Faculty of Business Administration, Southwestern University of Finance and Economics, Chengdu 610074, China; liyouyuan@swufe.edu.cn

**Keywords:** opinion dynamics, three-stage cascade, information attenuation, bounded confidence

## Abstract

In social network analysis, entropy quantifies the uncertainty or diversity of opinions, reflecting the complexity of opinion dynamics. To enhance the understanding of how opinions evolve, this study introduces a novel approach to modeling opinion dynamics in social networks by incorporating three-stage cascade information attenuation. Traditional models have often neglected the influence of second- and third-order neighbors and the attenuation of information as it propagates through a network. To correct this oversight, we redefine the interaction weights between individuals, taking into account the distance of opining, bounded confidence, and information attenuation. We propose two models of opinion dynamics using a three-stage cascade mechanism for information transmission, designed for environments with either a single or two subgroups of opinion leaders. These models capture the shifts in opinion distribution and entropy as information propagates and attenuates through the network. Through simulation experiments, we examine the ingredients influencing opinion dynamics. The results demonstrate that an increased presence of opinion leaders, coupled with a higher level of trust from their followers, significantly amplifies their influence. Furthermore, comparative experiments highlight the advantages of our proposed models, including rapid convergence, effective leadership influence, and robustness across different network structures.

## 1. Introduction

The proliferation of digital communication platforms, exemplified by WeChat, Weibo, and Facebook, has revolutionized the way individuals engage with one another. These platforms facilitate ubiquitous communication, enabling users to express and refine their viewpoints in real-time [1,2]. The dynamic nature of social interaction often involves individuals advocating for their perspectives and endeavoring to sway the decisions of their peers [3,4,5]. This phenomenon is particularly evident in the consumer decision-making process, where individuals frequently reassess their preferences based on the choices of their social circles, both in the physical and virtual realms [6]. Those who possess the capacity to significantly influence the opinions of others are recognized as opinion leaders [7]. In practical scenarios, these leaders often aim to steer public sentiment towards specific objectives [8].

The study of opinion dynamics within social networks has emerged as a critical area of research, focusing on the mechanisms through which opinions are formed and evolve among individuals under the influence of opinion leaders [9]. A user’s opinion is typically indicative of their stance on a given product or issue, and the field of opinion dynamics seeks to elucidate the intricate processes of opinion formation and consensus through the lens of individual interactions [10,11]. The landscape of opinion dynamics models is diverse, encompassing both discrete and continuous approaches. Common discrete models include the voting model [12], Sznajd model [13], and majority rule model [14]. For continuous opinions, the DeGroot (DG) model, a seminal linear model, was initially crafted to elucidate the formation of group consensus through interpersonal influence [15]. Subsequent refinements, such as the introduction of ‘rebels’ by Cao et al. (2018), have demonstrated that the consensus is contingent upon initial opinions and the structure of the learning environment [16]. Nonlinear models, particularly those predicated on bounded confidence, such as the Deffuant–Weisbuch (DW) and Hegselmann–Krause (HK) models, have garnered significant attention [17,18,19]. Lorenz (2010) further expanded the conceptual framework by developing a heterogeneous model that stratifies social groups based on varying confidence thresholds [20]. Within these groups, individuals are presumed to share similar confidence levels, whereas intergroup dynamics may involve a spectrum of confidence levels. Studies have posited that a heterogeneous HK model can achieve a state of equilibrium in collective opinion evolution, with the trajectory of group consensus being subject to nonlinear system analysis [21,22]. Contemporary opinion models have contributed valuable theoretical insights into the dynamics of public opinion, illustrating the nuanced interplay between individual interactions and opinion updates [23,24,25]. These models serve as a foundation for empirical research, shedding light on the complex interdependencies that shape public discourse.

Investigations into the determinants of opinion dynamics have garnered considerable scholarly attention. Research has underscored the pivotal roles of self-persistence, social influence, and the distribution of power within social structures in shaping the trajectory of individual opinion evolution [26]. For example, the role of stubborn agents has been widely studied in both voter models and linear consensus systems [27,28,29,30]. Dong et al. (2017) have delved into the pivotal role of leadership in the coalescence of consensus, leveraging the DG model to dissect its mechanisms [24]. Expanding on this, Ding et al. (2019) have scrutinized how the connectivity of network nodes and the propensity for self-affirmation can modulate the tempo at which consensus is achieved [31]. The disruptive impact of environmental perturbations on the crystallization of definitive opinions among opinion leaders and their followers has also been dissected by Zhao et al. (2016) [32]. It has been observed that opinion convergence is more pronounced in networks with random architectures compared to those with more structured topologies, highlighting the salient influence of network topology on opinion deadlocks [33,34]. Glass et al. (2020) have further posited that group size is a determinant of collective opinion uniformity, with obstinate factions potentially exerting a buffering effect on societal dynamics under certain conditions [35]. The salience of user-specific interests in steering opinion formation within social networks has also been underscored [36].

Despite these advancements, prevailing models of opinion dynamics have often neglected the aggregate influence of multiple tiers of network neighbors when modeling the likelihood of opinion propagation. Traditionally, these models have focused solely on the impact of direct neighbors. However, in the tapestry of real-world social networks, the opinions of an individual are not only swayed by their immediate social contacts but also by the more distant, yet influential, voices of their friends’ friends. Empirical studies have indicated that the collective influence of these extended social connections can surpass that of isolated nodes [37]. Moreover, the multiplicative effect of simultaneous interactions within a network can amplify the likelihood of opinion dissemination and foster a more nuanced evaluation of viewpoints [38]. Hence, there is a compelling need to integrate the multi-level cascade interactions within opinion dynamics models.

Another critical oversight in existing models is the disregard for information attenuation during opinion transmission. In the context of social networks, the relevance and persuasiveness of information are often attenuated as it traverses through the network, with closer nodes wielding greater influence over an individual’s opinion than those that are more distant. To address this, the development of opinion dynamics models that account for the decay of information as it propagates is imperative.

In this study, we introduce an HK inspired model of opinion dynamics that encapsulates a three-stage cascade mechanism of interaction. This model posits that as opinions evolve, agents retain a modicum of fidelity to their initial beliefs while recalibrating the influence of others based on the proximity of their opinions. This approach provides a more nuanced reflection of opinion evolution in social networks, grounded in the interplay between opinion dynamics, network theory, and the role of opinion leaders.

Our contribution lies in the three-level network of opinion transmission that integrates the concept of information attenuation. We propose two models of opinion dynamics under this cascade framework: one accommodating a unified opinion leader subgroup and the other considering two contending opinion leader factions. These models are designed to capture the influence of individual network nodes across three levels. Subsequently, simulation experiments are conducted to elucidate the dynamics of opinion evolution and improvements on existing methods.

The remainder of this study is organized as follows. The next section presents the basic concepts of graph theory and opinion dynamics. Section 3 presents the model of opinion transmission in a three-level network. Section 4 details and discusses the simulation results, and Section 5 provides concluding remarks.

## 2. Preliminaries

Prior studies have defined opinion leaders as individuals who control the flow of resources throughout a community [39]. Opinion leaders also have higher socioeconomic positions, which increase their close contact with individuals through increased exposure by mass media [40,41]. Opinion leaders exert a special influence over other individuals, and can influence the purchasing decisions of others by providing relevant suggestions about a product [42,43]. Therefore, opinion leaders mainly refer to definite, unwavering target opinions unaffected by other followers during the opinion update process. The direct intention of opinion leaders is to influence their followers [8]. In mass media, opinion leaders play an important role in the process of information diffusion [44]. In opinion dynamics, individuals classified as opinion leaders are recognized for their pivotal roles within the network, possessing the propensity to be readily adopted as influences [45]. The extant literature has consistently demonstrated the efficacy of these leaders in fostering consensus within social network structures [24,46]. Their influence is often attributed to their ability to articulate and propagate viewpoints that resonate with and galvanize the collective.

The dissemination of information represents the evolution of opinions in a trust communication network. Many studies have constructed opinion dynamics models based on the network topology [9,47]. Therefore, to describe the social impact between individuals, this study uses a communication network to model the coevolution of opinions.

A directed graph is a tuple *G* = *(*V, E*)*, where $V=\left\{V_{1},V_{2},\ldots V_{n}\;\right\}$ is the node of the set. $E=\left\{\left(i,j\right):i,j\in V\;\right\}\subset V\times V$ is the edge of the set. Individual i∈V affects individual j if and only if (i,j) ∈E. The edge is a path that joins a finite sequence of distinct nodes. If there is a path between paired nodes in the graph, they can be considered to be strongly connected.

During the progression of opinion formation, the field of opinion dynamics is concerned with the genesis and propagation of individual perspectives. Distinguished by their nature as either discrete or continuous, these opinions reflect the spectrum of individual beliefs within a social construct. Notably, the HK model captures the synchronous evolution of opinions within a group [18], while the DW model addresses the asynchronous aspect of opinion adjustment [17]. These paradigms are instrumental in deciphering the nuances of consensus formation and the conditions under which divergent viewpoints either find common ground or remain polarized.

Let $X(k)=\left\{x_{1}\left(k\right),x_{2}\left(k\right),\ldots \ldots x_{N}\left(k\right)\;\right\}$ be the opinion of individual *i* at time *k*. In bounded confidence models, for the case $\left|x_{i\;}\left(k\right)-x_{j\;}\left(k\right)\right|\leq \;\varepsilon$, the HK model can be described as follows:

(1) $$x_{i}\left(k+1\right)=\frac{\sum_{j:\left|x_{i\;}\left(k\right)-x_{j\;}\left(k\right)\right|\leq \varepsilon }\delta_{ij}x_{j}(k)}{\sum_{j:\left|x_{i\;}\left(k\right)-x_{j\;}\left(k\right)\right|\leq \varepsilon }\delta_{ij}},$$

where ε is the bounded confidence level of individual, and $\delta_{ij}$ is the weight that agent *i* assigns to agent *j* at time *t*. 

The interaction mechanisms of the DW and HK models are different, but the rules of these models are similar, with repeating under-bounded confidence. The DW model is

(2) $$\left\{\begin{array}{c} x_{i}\left(k+1\right)=x_{i}\left(k\right)+\eta \left[x_{j}\left(k\right)-x_{i}\left(k\right)\right]\\ x_{j}\left(k+1\right)=x_{j}\left(k\right)+\eta [x_{i}\left(k\right)-x_{j}\left(k\right)] \end{array},\right.$$

where η is the convergence parameter.

Based on the DW and HK models, various improved variants have been proposed. For instance, Zhao et al. (2016) present a bounded confidence-based opinion dynamics model that delineates the influence of opinion leaders and followers within a network [32]. The model segments social agents into two subgroups, opinion leaders with fixed target opinions and opinion followers with adjustable views, and simulates the group’s opinion evolution. It investigates the impact of the proportion of opinion leaders, the confidence levels of followers, and the trust degrees followers have in leaders, revealing that enhancing the credibility of opinion leaders is crucial for maximizing influence in e-commerce settings.

Within the framework of a bounded trust model, the communication of opinions among individuals is predicated on the difference in their initial beliefs falling within an acceptable threshold, defined by their confidence levels. This mechanism allows for interaction and potential convergence of viewpoints. Concurrently, the interplay of diverse confidence levels and the spectrum of initial opinions can lead to the emergence of three distinct collective opinion states: consensus, scattered opinions, and polarized opinions.

Gargiulo and Gandica (2017) introduce an agent-based model that integrates homophily in network morphogenesis to explore its impact on opinion dynamics, particularly focusing on the role of media presence [48]. The model demonstrates that homophily, contrary to common belief, fosters consensus rather than polarization. It extends the bounded confidence model by incorporating media influence, showing that media’s effectiveness diminishes under high pressure, and that homophily structures can significantly counteract media dominance by consolidating non-aligned opinions into a strong opposing cluster, thus potentially leading to societal opinion polarization.

## 3. The Proposed Methods

### 3.1. Information Attenuation in Three-Stage Cascade

Our model introduces information attenuation under a three-stage cascade architecture to analyze the influence of opinion leaders and the information flow within the network. In the context of a social network denoted as *G* = (*V*, *E*), where *V* represents a set of individuals partitioned into an opinion leaders set *L* and followers set *F*, and *E* symbolizes the set of edges, we introduce a weight matrix that encapsulates the bounded confidence for each individual dyad within the network.

Initially, considering that opinion leaders are not influenced by opinion followers, even if they are neighbors, we have redefined the adjacency matrix to circumvent this situation.

(3) $$a_{ij}=\left\{\begin{array}{cc} 1 & \left(i,j)\in E\;\mathrm{and}\;(i\in L\;\mathrm{or}\;j\in F\right)\\ 0 & \mathrm{otherwise}\;\; \end{array}\right.$$

*a_ij_* denotes the connectivity and the direction of information flow between individuals *i* and *j*. This parameter is instrumental in delineating the network’s structure and the pathways through which opinions are transmitted. Specifically, within this framework, opinion leaders are posited to be influenced exclusively by their peers among the opinion leaders themselves, establishing a closed loop of influence. In contrast, followers within the network are subject to a broader array of influences, being susceptible to persuasion from both opinion leaders and their fellow followers.

Then, a weight matrix $W=\{w_{ij}(t)\}$ is proposed, grounded in the principle of bounded confidence, which dictates the intensity of information transmission that one individual can exert over another based on their proximity of opinions. The weights are articulated as follows:

(4) $$w_{ij}(t)=\left\{\begin{array}{cc} a_{ij}\cdot e^{-\left|x_{i}(t)-x_{j}(t)\right|} & \left|x_{i}(t)-x_{j}(t)\right|\leq \varepsilon \\ 0 & \mathrm{otherwise} \end{array}\right.$$

*x_i_*(*t*) and *x_j_*(*t*) represent the opinions of individuals *i* and *j* at time *t*, and ε is the bounded confidence threshold. This threshold is pivotal as it determines the extent to which an individual is receptive to communication from another. If *j* is able to receive opinion information from *i*, the closer their opinions are, the closer *w_ij_* approaches one, indicating a greater influence exerted by *i* on *j*. Conversely, the greater the divergence in their opinions, the closer *w_ij_* approaches 0. Additionally, considering the bounded confidence, once $w_{ij}(t)<e^{-\varepsilon }$, it equals 0.

In the realm of opinion dynamics, the influence on an individual extends beyond their immediate opinion neighbors to encompass the broader network of influence, including second-order neighbors (those connected via an intermediary) and third-order neighbors (those reached through two degrees of separation). If *i* needs to transmit opinion information to *j* through an intermediary individual *k*, then the influence of *i* on *j* can be represented by a composite weight $w_{ik}(t)\cdot w_{kj}(t)$, which accounts for the multi-stage interactions within the network.

However, the fidelity of information during transmission is not absolute, leading to some degree of information loss. Due to intermediaries intentionally or unintentionally concealing or altering information, the actual strength of influence from *i* to *j* through *k* should be lower than the aforementioned composite weight. Thus, information transmission is subject to attenuation, which must be incorporated into the model. The information attenuation depends on two factors. First, it is directly proportional to the number of intermediaries in the transmission path; more individuals in the transmission path lead to greater information attenuation. Second, the strength of influence along the transmission path affects attenuation. For instance, the smaller the composite weight $w_{ik}(t)\cdot w_{kj}(t)$, the more severe the attenuation of information transmitted from *i* to *j* through *k*. This is because hesitation or distrust among individuals in the transmission path increases the likelihood of information distortion. 

Since the composite weight on any path is always less than or equal to 1, we propose a three-stage cascade weight that takes into account information attenuation. In this model, ${\left[w_{ik}(t)\cdot w_{kj}(t)\right]}^{2}$ and ${\left[w_{ik}(t)\cdot w_{ks}(t)\cdot w_{sj}(t)\right]}^{3}$ are used to replace the corresponding composite weight; the differences reflect the information attenuation along the transmission path. 

The evolution of an individual’s opinion is contingent upon the most compelling pathway of information transmission. We update the weight matrix as $\bar{W}(t)$, which is designed to account for the intricacies of three-stage cascade formation attenuation:

(5) $$\begin{array}{ll} {\bar{w}}_{ij}(t)= & \underset{k,s=1,2,\ldots ,N}{\max}\;\eta \\ s.t.\;\;\eta = & \max\{\eta_{1},\eta_{2},\eta_{3}\}\\ \eta_{1}= & w_{ij}(t)\\ \eta_{2}= & {\left[w_{ik}(t)\cdot w_{kj}(t)\right]}^{2}\\ \eta_{3}= & {\left[w_{ik}(t)\cdot w_{ks}(t)\cdot w_{sj}(t)\right]}^{3} \end{array}$$

**Theorem 1.**  *If a_ij_* = 1, *then* ${\bar{w}}_{ij}(t)=w_{ij}(t)$.

**Proof of Theorem 1.**  We first compare the relative magnitudes of $\eta_{1}$ and $\eta_{2}$.

**Figure 1:**
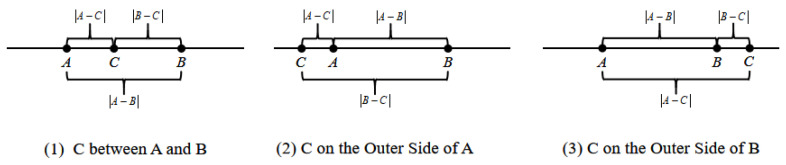
Illustrations of three points A, B, and C.

As shown in Figure 1, it can be proven that for any three points ‘A, B, C’ on a number line, regardless of their arrangement, the following conclusion holds:

(6) $$\left|A-C\right|+\left|C-B\right|\geq \left|A-B\right|$$

That is,

(7) $$\left|x_{i}(t)-x_{k}(t)\right|+\left|x_{k}(t)-x_{j}(t)\right|\geq \left|x_{i}(t)-x_{j}(t)\right|$$

(8) $$e^{-(\left|x_{i}(t)-x_{k}(t)\right|+\left|x_{k}(t)-x_{j}(t)\right|)}\leq e^{-\left|x_{i}(t)-x_{j}(t)\right|}$$

If *a_ik_* = *a_kj_* = 1,

(9) $$w_{ik}(t)\cdot w_{kj}(t)\leq w_{ij}(t)$$

If *a_ik_* = 0 or *a_kj_* = 0, since *a_ij_* = 1, the inequality mentioned above still holds true.

Because $w_{ik}(t)\cdot w_{kj}(t)\leq 1$,

(10) $$\eta_{2}={\left[w_{ik}(t)\cdot w_{kj}(t)\right]}^{2}\leq w_{ij}(t)=\eta_{1}$$

By similar reasoning, it can be proven that $\eta_{3}\leq \eta_{1}$.

In summary, as long as *a_ij_* = 1, regardless of how *k* and *s* vary, it is guaranteed that ${\bar{w}}_{ij}(t)=w_{ij}(t)$. □

Theorem 1 demonstrates that if node *i* in a social network can directly convey an opinion to node *j*, satisfying both connectivity and bounded confidence, then their information transmission is not influenced by any intermediary nodes. In other words, the three-stage cascade model only applies to situations where direct information transmission is not feasible, i.e., *a_ij_* ≠ 1, serving as a complement to traditional opinion dynamics models.

Finally, the weights are normalized as follows:

(11) $${\hat{w}}_{ij}(t)=\frac{{\bar{w}}_{ij}(t)}{\sum_{i}{\bar{w}}_{ij}(t)}$$

### 3.2. Model I: Single Opinion Leader Subgroup

There are *N* agents {*c*_1_, *c*_2_, …, *c_N_*} assumed in a social network, including *N*1 opinion leaders {*c*_1_, *c*_2_, …, *c_N_*_1_} and *N − N*1 followers {*c_N_*_1+1_, *c_N_*_1+2_, …, *c_N_*}.

The opinion updating model for the leader subgroup is proposed as follows:

(12) $$x_{i}^{L}(t+1)=(1-\lambda_{i})\left[\beta_{i}x_{i}^{L}(t)+(1-\beta_{i})\frac{1}{N_{i}^{L}(t)}\sum_{j=1}^{N1}{\hat{w}}_{ji}(t)x_{j}^{L}(t)\right]+\lambda_{i}d$$

for *i* = 1, 2, …, *N*1, where $N_{i}^{L}(t)=\sum_{j=1}^{N1}{\hat{w}}_{ji}(t)$.

$x_{i}^{L}(t)$ refers to the opinion of the *i*th leader, *d* is the value of the target opinion of the opinion leader subgroup, and $\lambda_{i}$ is the influence intensity of target opinion *d*. $\beta_{i}$∈[0,1] is the degree of self-confidence of agent *i*.

The opinion-updating model for the follower subgroup is proposed as follows:

(13) $$x_{i}^{F}(t+1)=\beta_{i}x_{i}^{F}(t)+\alpha_{i}\frac{1}{N_{i}^{L}(t)}\sum_{j=1}^{N1}{\hat{w}}_{ji}(t)x_{j}^{L}(t)+(1-\alpha_{i}-\beta_{i})\frac{1}{N_{i}^{F}(t)}\sum_{j=N1+1}^{N}{\hat{w}}_{ji}(t)x_{j}^{F}(t)$$

where $N_{i}^{F}(t)=\sum_{j=N1+1}^{N}{\hat{w}}_{ji}(t)$. $x_{i}^{F}(t)$ is the opinion of the *i*th agent belonging to the follower subgroup. $\alpha_{i}$∈[0,1] is the trust level of the agent *i* towards the leaders.

### 3.3. Model II: Two Competitive Opinion Leader Subgroups

There are two opinion leader subgroups, leader subgroup 1 and leader subgroup 2, which have competitive target opinions. Assume *N* agents {*c*_1_, *c*_2_, …, *c_N_*} in a social network, *N*1 is the number of leaders of subgroup 1 {*c*_1_, *c*_2_, …, *c_N_*_1_}, *N*2 is the number of leaders of subgroup 2 {*c_N_*_1+1_, *c_N_*_1+2_, …, *c_N_*_1*+N*2_}, and *N − N*1 *− N*2 opinion followers {*c_N_*_1*+N*2*+*1_, *c_N_*_1*+N*2*+*2_, …, *c_N_*}. 

The opinion-updating model for leader subgroup 1 is proposed as follows:

(14) $$x_{i}^{L1}(t+1)=(1-\lambda_{i})\left[\beta_{i}x_{i}^{L1}(t)+(1-\beta_{i})\frac{1}{N_{i}^{L1}(t)}\sum_{j=1}^{N1}{\hat{w}}_{ji}(t)x_{j}^{L1}(t)\right]+\lambda_{i}d_{1}$$

for *i* = 1, 2, …, *N*1, where $N_{i}^{L1}(t)=\sum_{j=1}^{N1}{\hat{w}}_{ji}(t)$ and *d*_1_ is the target opinion of the first leader subgroup.

The opinion-updating model for leader subgroup 2 is proposed as follows:

(15) $$x_{i}^{L2}(t+1)=(1-\lambda_{i})\left[\beta_{i}x_{i}^{L2}(t)+(1-\beta_{i})\frac{1}{N_{i}^{L2}(t)}\sum_{j=N1+1}^{N1+N2}{\hat{w}}_{ji}(t)x_{j}^{L2}(t)\right]+\lambda_{i}d_{2}$$

for *i* = *N*1 *+* 1, *N*1 *+* 2, …, *N*1 *+ N*2, where $N_{i}^{L2}(t)=\sum_{j=N1+1}^{N1+N2}{\hat{w}}_{ji}(t)$ and *d*_2_ is the target opinion of the second leader subgroup.

The opinion-updating model for the follower subgroup is proposed as follows:

(16) $$\begin{array}{ll} x_{i}^{F}(t+1)= & \beta_{i}x_{i}^{F}(t)+\alpha_{i}\frac{1}{N_{i}^{L1}(t)+N_{i}^{L2}(t)}(\sum_{j=1}^{N1}{\hat{w}}_{ji}(t)x_{j}^{L1}(t)+\sum_{j=N1+1}^{N1+N2}{\hat{w}}_{ji}(t)x_{j}^{L2}(t))\\ & +(1-\alpha_{i}-\beta_{i})\frac{1}{N_{i}^{F}(t)}\sum_{j=N1+N2+1}^{N}{\hat{w}}_{ji}(t)x_{j}^{F}(t) \end{array}$$

for *i* = *N*1 *+ N*2 *+* 1, *N*1 *+ N*2 *+* 2, …, *N*, where $N_{i}^{F}(t)=\sum_{j=N1+N2+1}^{N}{\hat{w}}_{ji}(t)$.

## 4. Simulation Experiments

### 4.1. Initial Conditions

We designed a suite of simulation experiments aimed at dissecting the opinion dynamics models within the framework of a three-stage cascade information transmission mechanism. The experimental design initiates with the following foundational parameters:

(1) An Erdős–Rényi random graph model G (*n*, *p*), with fixed nodes *n* and edge probabilities *p*, was used to generate a random network [49]. The generation process of the G(*n*, *p*) random graph involves initializing a set of *n* nodes and iteratively determining the presence or absence of edges between each pair of nodes based on a fixed connection probability *p*. Specifically, for each pair of distinct nodes (*i*, *j*), where *i* < *j*, a uniform random number *r* ∈ [0, 1] is generated. If *r* is less than *p*, an edge is placed between nodes *i* and *j*. This process is repeated for all *n*(*n* − 1)/2 possible pairs of nodes, ultimately yielding a random graph with *n* nodes and a probability *p* of connection between any two nodes. In this experiment, the probability *p* of connecting two nodes was set to 0.4.
(2) The number of nodes in this random network was 200. In Model I, there were 10 opinion leaders and 190 followers. In Model II, there were 10 opinion leaders for each target opinion, and the number of followers was 180.
(3) Assume that the leader subgroup’s target opinion in Model I is 1. The two competitive opinion leader subgroups have opposite target opinion values, i.e., 1 and −1.
(4) The initial opinions of leaders and followers are uniformly distributed. Individuals have initial opinions within the interval [0, 1] in Model I and within [−1, 1] in Model II.
(5) The threshold of confidence level ε in the bounded confidence is set as 0.5.
(6) The influence intensity of the target opinion for opinion leader i is $\lambda_{i}=0.5$.
(7) For individual *i* (*i* = 1, 2, …, *N*), the degrees of self-confidence and trust in the leader subgroup(s) are set as $\beta_{i}=0.4$ and $\alpha_{i}=0.4$.

Based on the above initial conditions, we analyzed the changes in four experimental parameters to explore the impact of various ingredients on the results. And the proposed cascade model was compared with other opinion dynamics models and on various types of random networks.

### 4.2. Parametric Analysis: Number of Opinion Leaders

**Figure 2:**
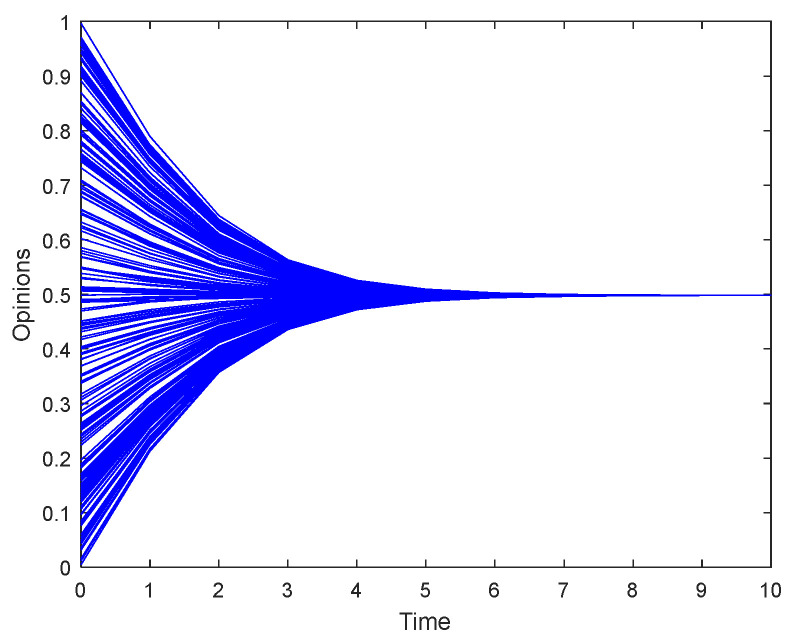
Opinion evolution of followers without a leader.

Firstly, we consider a leaderless scenario where opinions spread among followers. We set the number of leaders in the model to 0 and observed the evolution of agents’ opinions as shown in Figure 2, where each blue line represents the opinion of a follower. It can be seen that without the influence of opinion leaders, followers’ opinions tend towards the average initial opinion of the group. Therefore, the lack of leadership will lead to the homogenization of opinions.

**Figure 3:**
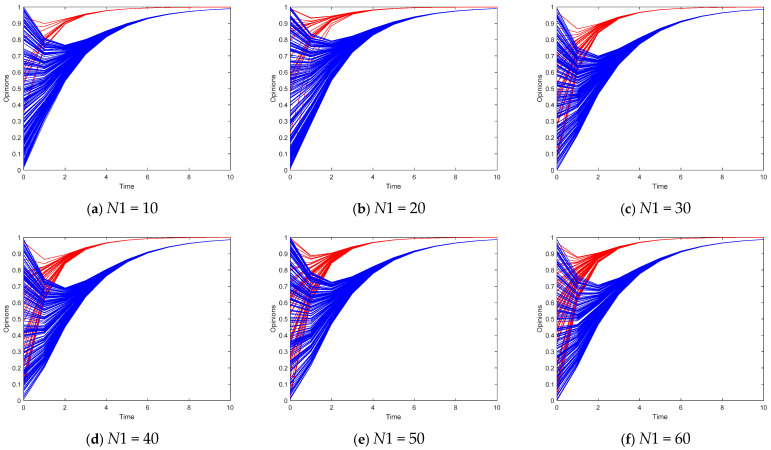
Opinion evolution in Model I with different *N*1.

Secondly, we explore scenarios with leaders. Figure 3 illustrates the changing number of opinion leaders in Model I and the evolution of the collective opinions of opinion leaders and followers. The red and blue solid lines represent the opinion leaders and followers, respectively. Regardless of the number of leaders, the leader’s opinion can quickly converge toward the target opinion of the leader subgroup in fewer than six time steps, whereas the opinions of the followers require more time to reach a stable consensus. Compared to the leaderless scenario, agents’ opinions are biased by the leader’s target opinion, rather than approaching the average opinion. However, we can also see that even when the number of opinion leaders increases, it has no obvious influence on the opinion followers.

For Model II, we alter the number of leaders in subgroups 1 and 2 to analyze the evolution of the followers’ opinions. The number of leaders in subgroups 1 and 2 are increased to 20, 30, 40, and 50 within the 200 nodes. To explicitly demonstrate the impact of the change in the number of the leaders of subgroups 1 and 2 on the opinions of the followers in Model II, Table 1 presents the distribution of the followers. The first row indicates the number of opinion leaders in subgroup 2, and the first column indicates the number of leaders in subgroup 1. The elements in Table 1 are the distribution of the followers with positive (>0.5), neutral ([−0.5, 0.5]), and negative opinions (<−0.5) at *t* = 10. As the number of leaders in subgroup 1 increases, the proportion of positive opinion followers increases and that of negative opinion followers decreases. As the number of leaders in subgroup 2 increases, the proportion of negative opinion followers increases and that of positive opinion followers decreases. The results show that the number of opinion leaders has an important influence on the distribution of followers’ opinions.

### 4.3. Parametric Analysis: Confidence Level Threshold

The initial threshold of the confidence level in both Model I and Model II was 0.1. We altered the threshold of the confidence level to demonstrate the impact of such changes on the evolution of the collective opinion of the leaders and followers.

**Figure 4:**
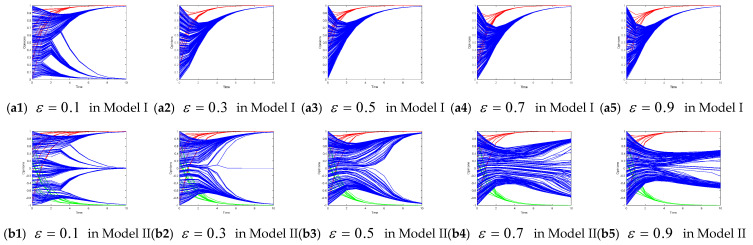
Opinion evolution with different confidence levels.

Figure 4 shows the evolution of the collective of the leaders’ and followers’ opinions when the confidence level threshold increases from 0.1 to 0.9 in Models I and II. In Model I, as the confidence level threshold increases, the opinions of leaders quickly converge toward positive opinions in fewer than six time steps. However, the opinion evolution of the opinion followers is complicated. When ε = 0.1, the opinion of most followers converges toward a positive opinion, and a few of the followers converge on the neutral opinion. When ε is 0.3, 0.5, 0.7, or 0.9, the follower’s opinion converges on a positive opinion. The simulation results of Model I reveal that when ε is very small (0.1), the standard of bounded confidence is very strict, and individuals do not accept others’ opinions, which depends to a large extent on the initial opinion. When ε is larger (0.3, 0.5, 0.7, or 0.9), the change in the confidence level threshold has no obvious influence on the opinions of the leaders or the followers.

In Model II, as the confidence level threshold increases, the opinions of the opinion leaders in both subgroups 1 and 2 converge toward the target opinion of the corresponding subgroup (1 or −1) in fewer than six time steps. The opinion evolution among the opinion followers is more complicated. When ε = 0.1 or ε = 0.3, the final opinions of followers are divided into three clusters with opinion values of −1, 0, and 1, showing a polarization phenomenon. When ε = 0.5, the followers’ opinions converge toward the target opinion of the corresponding subgroup (1 or −1). When ε = 0.7 or ε = 0.9, the final opinions of followers are scattered within [−0.8, 0.8], and no consensus is reached until after 10 iterations. The simulation results of Model II reveal that when ε is very small (0.1 or 0.3), the final follower’s opinion largely depends on the initial opinion. When ε is very large (0.7 or 0.9), the standard of bounded confidence is weak, individuals are willing to accept all aspects of opinions, and most of the followers’ opinions converge to the neutral opinion. When ε = 0.5, the followers’ opinions tend to converge to the target opinion of one of the opinion leaders (1 or −1).

### 4.4. Parametric Analysis: Influence Intensity of the Target Opinion

**Figure 5:**
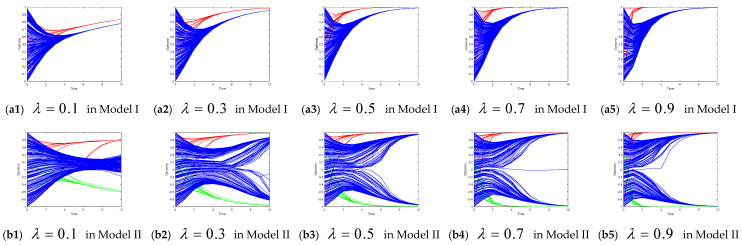
Opinion evolution under different intensities of the target opinion.

Figure 5 shows the evolution of leader and follower opinions when the intensity of the target opinion increases from 0.1 to 0.9. In Model 1, as the intensity of the target opinion increases, the time required for the leader to reach a consensus decreases, and the leader and followers’ opinions converge to the target opinion faster.

In Model II, as the intensity of the target opinion increase, the time required for the opinion leaders of subgroups 1 and 2 to converge to the target opinion of the corresponding subgroup decreases. When λ = 0.1, the leaders’ opinions do not reach a consensus on the target after 10 iterations. The leaders of subgroup 1 converge on an opinion of 0.8, and the leaders of subgroup 2 converge on 0.6. When λ = 0.3, 0.5, 0.7, or 0.9, the opinions of the leaders of subgroups 1 and 2 converge toward the target opinion of the subgroup (−1 or 1). The opinion evolution among opinion followers is more complicated. When λ = 0.1, the followers’ final opinions are scattered within [−0.2, 0.4], and no consensus is reached. When λ = 0.3, the followers’ final opinions are scattered within [−0.8, 0.2] and [0.6, 0.8]. When λ = 0.5, the followers’ final opinions converge on opinion −1 or 1. When λ = 0.7, the followers’ final opinions are divided into three clusters with −1, 0, and 1. When λ = 0.9, the followers’ opinions quickly converge on opinions −1 or 1.

These results indicate that when λ is very small (e.g., 0.1), the influence of the target opinion (such as advertising) is weak. The opinion leaders and followers slowly reach the target opinions, and the opinion convergence speed of opinion leaders and followers accelerates. When λ is larger, some opinion followers cannot follow the opinion leaders’ evolution over time. Limited by the principle of bounded confidence of opinions, they do not accept (or distrust) other individuals who approach the target opinion quickly, such as in the case of λ=0.7, and there are people who have a neutral opinion in the results of Model II.

### 4.5. Parametric Analysis: Degree of Self-Confidence and Trust in Opinion Leaders

**Figure 6:**
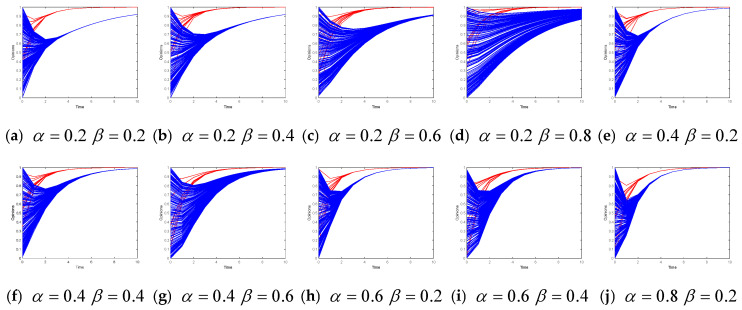
Opinion evolution under different self-confidence and trust levels in Model I.

**Figure 7:**
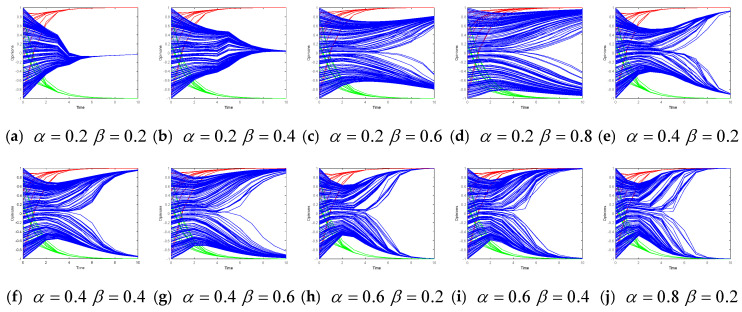
Opinion evolution under different self-confidence and trust levels in Model II.

In Figure 6 and Figure 7, α is the degree of trust placed by opinion followers in the opinion leaders, and β is the degree of the opinion followers’ self-confidence. Furthermore, these figures illustrate the evolution of collective opinions when α and β vary in Models I and II, respectively.

It can be seen from Figure 6 that with the increase in trust and self-confidence, the opinion leaders’ opinions quickly converge to opinion 1 in a short period of time. As trust in opinion leaders increases, followers’ opinions converge to target opinions more quickly. However, as the self-confidence level increases, the convergence to the target opinion becomes slower.

Figure 7 shows that as the trust and self-confidence increase, the opinion of the leaders in subgroups 1 and 2 rapidly converge to the target opinion subgroup (1 or −1) in fewer than six time steps. As the trust in the two subgroups of opinion leaders continues to increase, the followers’ opinions converge faster to the target opinion of the corresponding opinion leaders. However, as their self-confidence increases, the followers’ opinion convergence to the target opinion becomes slower.

In summary, as the opinion followers’ degrees of trust in leaders increase, followers are more willing to follow leaders’ opinions. As the opinion followers’ degrees of self-confidence increase, followers tend to maintain their original opinion, and the convergence speed decreases.

### 4.6. Comparison on Various Types of Random Networks

Generally, network structure is an important factor affecting the evolution of opinions. The proposed model was observed on various types of random networks. In addition to Erdős–Rényi random graphs, small-world networks and scale-free networks are also common. Furthermore, a growing network model proposed in Gargiulo and Gandica (2017), with different homophily levels, was also involved in the comparative experiment to explore the results for random networks with high cluster coefficients. The scale-free network defines the probability of a node being linked by a new node based on its degree, and the growing network adds the similarity of node opinions. A parameter *beta* is used to represent the coefficient tuning the homologous effect. Research has shown that modular increases with *beta*, meaning that more significant community structures (i.e., clusters) are formed [48].

In terms of parameter selection in the experiment, the number of nodes included in various networks was still 200. After examining the initially generated Erdős–Rényi random graph, we set two parameters in the small-world network generation process: the number of neighbors per node to 10, in order to maintain similarity to the average number of neighbors in the initial random network, and the rewiring probability to 0.1, 0.5, and 0.9, respectively, to observe the opinion dynamics on the network structure under different parameters. The generation process of both scale-free networks and the growing network depends on the Barabási–Albert model [50], where the parameters involved follow the settings in [48]: the number of initial core is 5, the degree distribution is 3, and the homophily coefficient is 5, 20 and 100. The degree distribution refers to the number of edges connected to each node. Furthermore, in the scale-free network, more parameters values for the degree distribution (3, 10, 30) were considered to observe the impact of network structure changes on opinion dynamics. All other parameter settings in the opinion dynamics model remained unchanged from the initial conditions introduced in Section 4.1.

**Figure 8:**
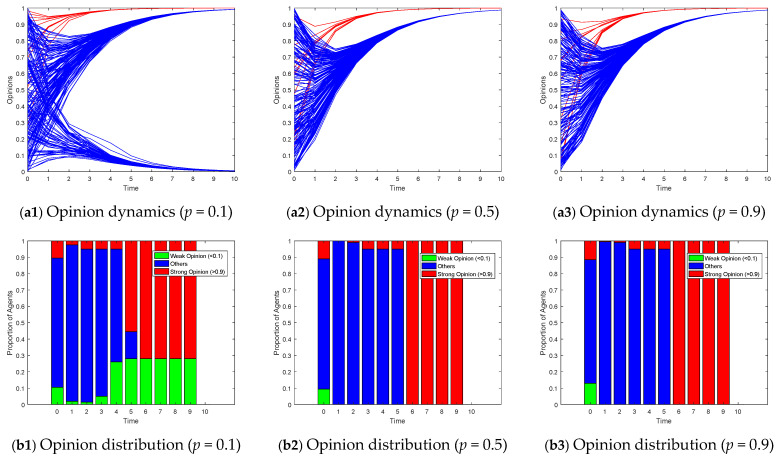
Model I on small-world networks with the different rewiring probability *p*.

**Figure 9:**
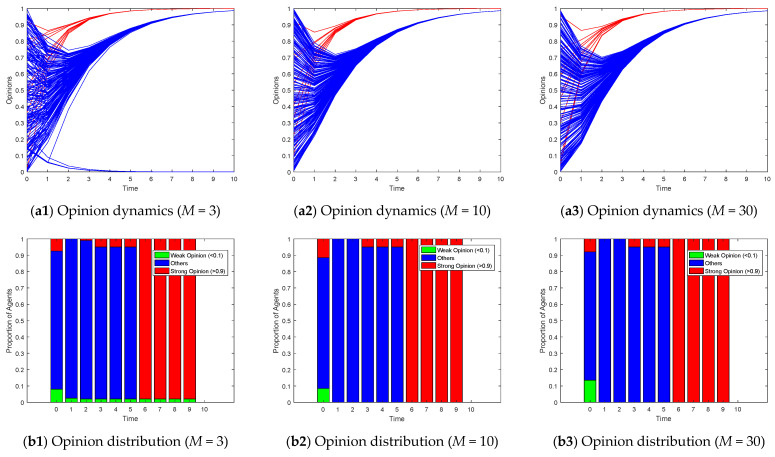
Model I on scale-free networks with the different degree distribution *M*.

**Figure 10:**
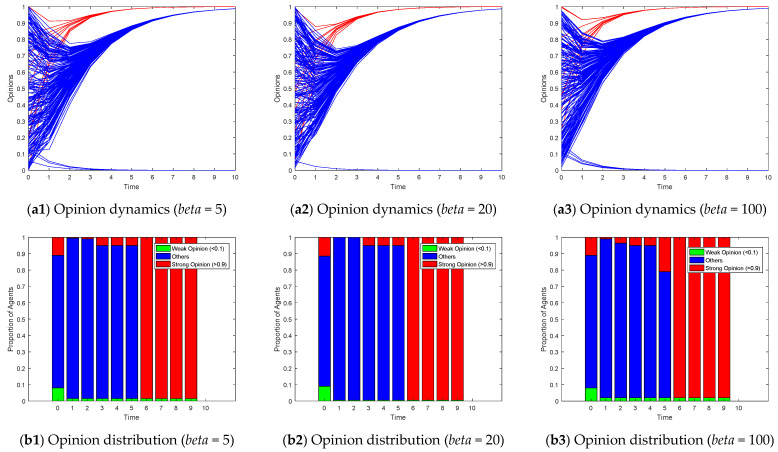
Model I on growing networks with the different homophily coefficient *beta*.

Figure 8, Figure 9 and Figure 10 illustrate the opinion evolution across various random network types using the proposed Model I. The performance of the model is assessed in terms of how effectively it guides the opinions of followers towards the target opinions of opinion leaders. To clearly illustrate the changes in the distribution of opinions over time, we depicted the opinion distribution in each experiment using stacked bar charts. Since Model I describes scenarios with a single leader group and opinions ranging from 0 to 1, we aimed to understand the degree of opinion polarization. Therefore, we defined agents with opinion values greater than 0.9 as holding strong opinions, represented by red bars indicating their proportion; agents with opinion values less than 0.1 as holding weak opinions, represented by green bars indicating their proportion; and the remaining agents with intermediate opinion values were represented by blue bars. By examining the model’s efficacy in guiding opinion dynamics, we can draw insights into the influence of network topology on opinion formation and the role of opinion leaders in these processes.

In small-world networks, when the rewiring probability is small, the edges in the network largely maintain their original regular connection patterns, with only a few edges being rewired. In this case, the structural characteristics of the network are closer to those of a regular network, exhibiting a high clustering coefficient and a longer shortest path length. As the rewiring probability increases, more and more edges are randomly rewired, leading to gradual changes in the network’s structural characteristics. When the rewiring probability approaches 1, the edges in the network are almost completely randomly rewired, at which point the network’s structural characteristics are closer to those of a random network, characterized by a shorter shortest path length and a lower clustering coefficient. As shown in Figure 8(a1), when a small-world network exhibits a high clustering coefficient, nodes within the network form tight clusters, indicating that the connections between nodes are very dense. This allows information to spread rapidly within these clusters, and their opinions become reinforced within the group, gradually evolving into divergent opinions. However, as the rewiring probability increases, e.g., *p* = 0.5 and *p* = 0.9 in Figure 8, the network structure acquires characteristics of random networks, leading to a convergence of group opinions towards a consensus on the leaders’ target.

Figure 9 illustrates the impact of varying the degree distribution (*M*) in a scale-free network on opinion dynamics. Scale-free networks are characterized by a power-law degree distribution, where a few nodes have a large number of connections, while most nodes have relatively few. When the degree distribution is low (e.g., *M* = 3 in Figure 9), there are fewer highly connected nodes in the network. This limits the speed at which information can propagate, resulting in slower opinion convergence and possibly the formation of multiple opinion clusters. As the degree distribution increases (e.g., *M* = 10 and *M* = 30), the number of highly connected nodes grows. These hub nodes act as efficient conduits for information dissemination, accelerating the propagation of opinions and facilitating faster convergence towards the leaders’ target opinions.

Figure 10 explores the effects of altering the homophily coefficient (*beta*) in a growing network model on opinion evolution. Growing network models simulate the gradual addition of new nodes to the network, often with a preference for connecting to similar nodes (homophily). According to the parameter settings of our experiment, the scale-free network constructed when *M* = 3 can be regarded as a special case of setting *beta* to 0. At low homophily levels (e.g., *beta* = 5 in Figure 10), new nodes are less discriminatory in their choice of connections, leading to a more random mix of node interactions. This can slow down the convergence of opinions as information diffuses more broadly across the network. As the homophily coefficient increases (e.g., *beta* = 20 and *beta* = 100), new nodes become more selective, preferring to connect with nodes that hold similar opinions. This tends to reinforce existing opinion clusters, potentially accelerating opinion convergence within those clusters. However, unlike the results observed in small-world networks, the increasing homophily in the growing network model does not lead to significant opinion polarization or fragmentation. The opinions of the vast majority of agents tend to converge towards the target opinions of leaders, although they may converge at different speeds depending on the strength of homophily. Moreover, the evolution of opinions did not show significant differences with changes in homophily.

**Figure 11:**
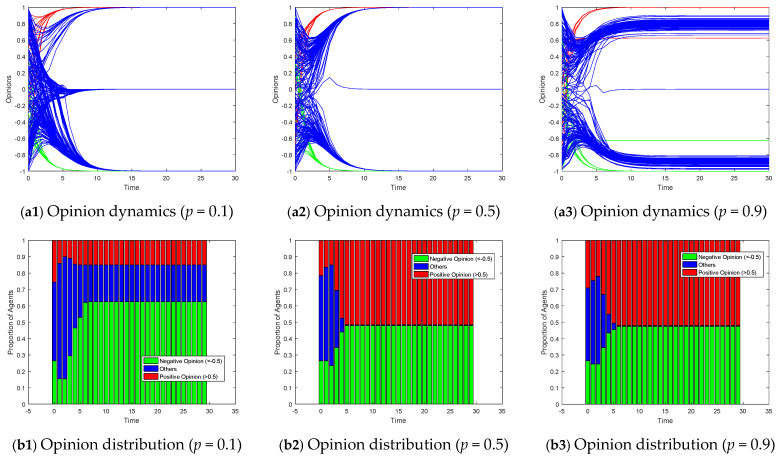
Model II on small-world network with the different rewiring probability *p*.

**Figure 12:**
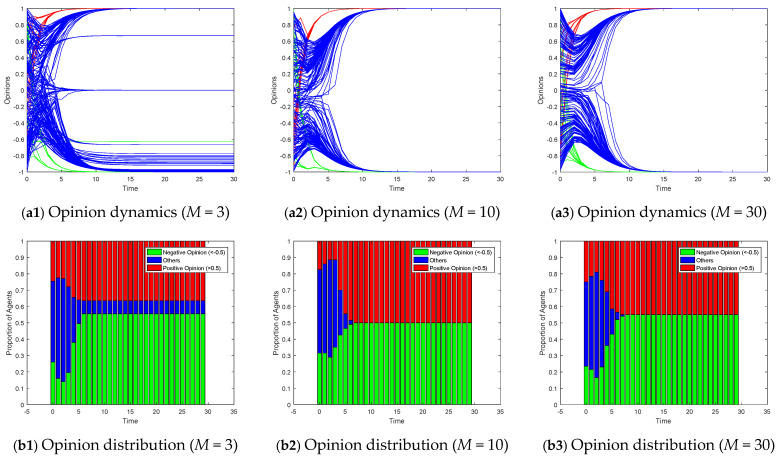
Model II on scale-free networks with the different degree distribution *M*.

**Figure 13:**
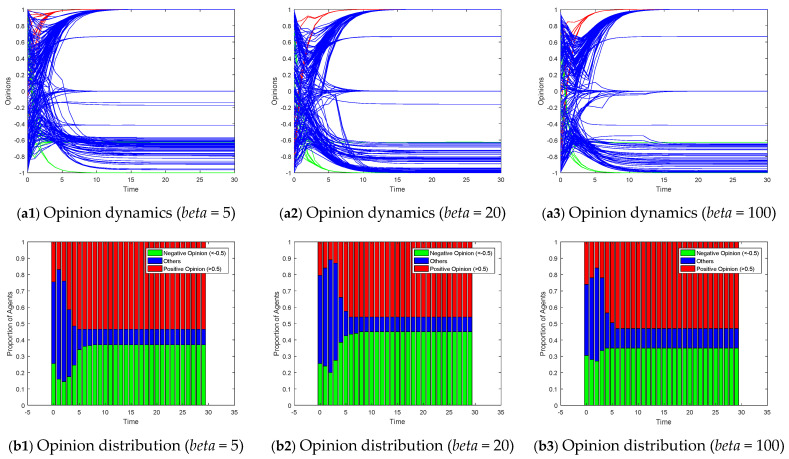
Model II on growing networks with the different homophily coefficient *beta*.

For Model II, considering the scenario of two competing subgroups of opinion leaders over the interval [−1, 1], we employ a stacked bar chart to depict the proportions of positive (opinion values > 0.5) and negative (opinion values < −0.5) opinions, thereby illustrating the degree of polarization. Figure 11 exhibits the opinion dynamics and opinion distributions within small-world networks under varying rewiring probabilities for Model II. In highly clustered scenarios (*p* = 0.1), opinions within the network tend to evolve around the opinions within each cluster. When no opinion leaders exist within a cluster, it degenerates into a leaderless situation, converging towards a neutral opinion. As the rewiring probability increases, the network transitions from a highly clustered structure to a more random one, facilitating the spread of targeted opinions throughout the network. Consequently, the proportion of individuals converging to neutral opinions decreases, revealing more pronounced signs of polarization. Figure 12 displays the impact of varying the degree distribution in a scale-free network on the opinion dynamics in Model II. In networks with a low degree distribution (*M* = 3), the number of highly connected nodes is limited, leading to slower opinion convergence and more agents holding a neutral opinion. As the degree distribution increases, the presence of hub nodes facilitates faster dissemination of opinions, resulting in quicker alignment of follower opinions towards the target opinions of the leader subgroups. Figure 13 explores the effects of altering the homophily coefficient in a growing network model on the opinion dynamics in Model II. Consistent with the discussion on Figure 10, changes in the homophily coefficient do not significantly alter the opinion polarization.

Overall, networks with high clustering or homophily tend to exhibit rapid opinion convergence and polarization, and traditional models show significant differences in opinion evolution on these networks. However, the proposed model’s consideration of up to third-order neighbors significantly expands the potential range of influence an opinion leader can exert. This extended reach means that opinions are not solely dictated by direct connections but are also shaped by more distant relational pathways. As a result, the model exhibits a level of robustness against the structural idiosyncrasies of different network types. It provides a more stable and consistent prediction of opinion dynamics, which is valuable for applications where network structure may be unpredictable or subject to change.

### 4.7. Comparison with Other Opinion Dynamics Models

**Figure 14:**
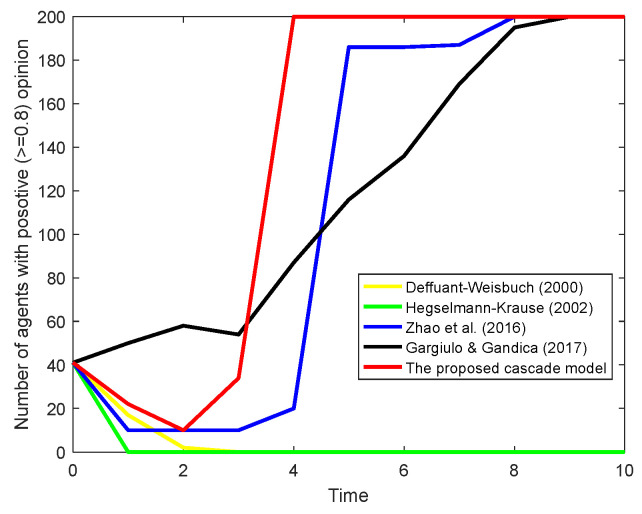
Comparative experimental results for single leader group [17,18,32,48].

Based on the initial settings of the experiment, the model in this paper is compared with other continuous opinion dynamics models. Figure 14 presents a comparative analysis of the proposed three-stage cascade information attenuation model against other established opinion dynamics models, specifically focusing on scenarios with a single opinion leader subgroup.

In the compared models, the DW and HK models are implemented using Equations (1) and (2) introduced in Section 2, where the parameters ε = 0.5, $\delta_{ij}\;$= 1 and η = 0.6. The models proposed in [32] and [48] are implemented using the formulas and parameters introduced in the corresponding article, respectively.

The DW and HK models, lacking a mechanism to incorporate opinion leaders, show a general trend in positive opinions converging towards disappearance over time. This suggests that without directed leadership, the network’s opinion dynamics tend to the average initial opinion without a distinct directionality, which is consistent with the results shown in Figure 2. The model in [32], which includes opinion leaders, demonstrates a more pronounced shift towards the leader’s opinion, especially after a few time steps. It shows that the presence of a leader can significantly influence the network towards a consensus that aligns closely with the leader’s opinion. Although the method proposed by [48] does not involve the role of opinion leaders, it takes into account the positive impact of the presence of media on public opinion, resulting in the population holding positive opinions growing at an approximately linear rate in Figure 14.

Significantly different from the traditional methods mentioned above, our proposed model exhibits a rapid convergence towards the opinion leader’s stance within a very short timeframe. This swift alignment is attributed to the model’s ability to consider information dissemination across three stages of interaction, which allows for a more nuanced and effective propagation of influence. The model not only captures the direct influence of the leader but also the moderated influence through second- and third-order connections, leading to a quicker and more unified consensus towards the leader’s opinion.

The results show that the model facilitates faster opinion convergence, which is crucial in environments where timely opinion alignment is necessary, such as during public crises or rapid information dissemination campaigns.

**Figure 15:**
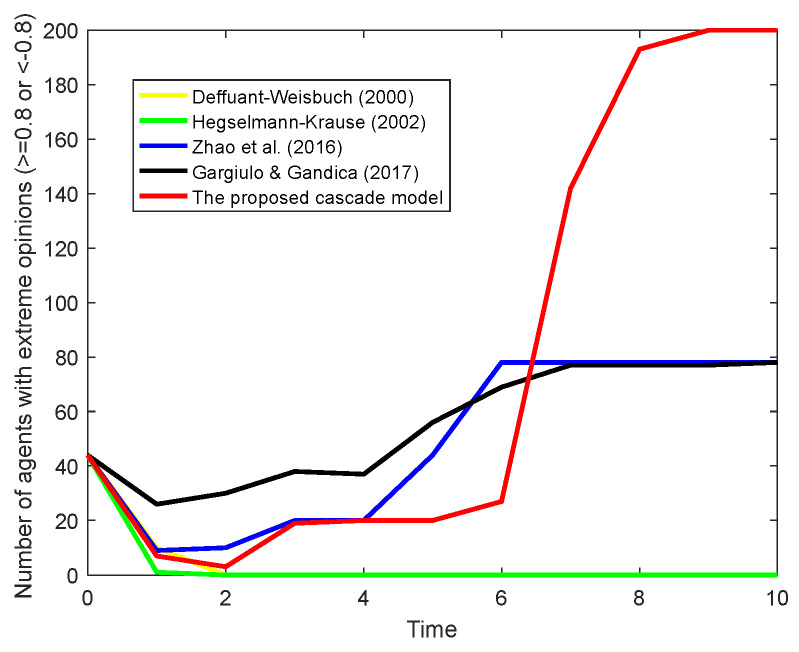
Comparative experiment results for two leader groups [17,18,32,48].

Figure 15 extends the comparative analysis to scenarios where two competing opinion leader subgroups are present within the network. The number of agents with extreme opinions, i.e., their opinions are not less than 0.8 or less than −0.8, is observed to highlight how the network’s opinion dynamics evolve under the influence of opposing leaders. Except for agents who hold extreme opinions, other nodes in the network are neutral. As shown in Figure 15, in the DW and HK models, the agents’ opinions tend to neutralize without significant alignment towards any particular stance. In contrast, the [32] and [48] models demonstrate a more pronounced polarization of opinions. However, there are still more than half of the agents who have not followed the opinions of any leaders, thus remaining neutral.

However, it is the proposed cascade model that stands out, showing the quickest and most decisive impact on opinion polarization. By considering the three-stage cascade of influence and information attenuation, the model not only captures the direct influence of the leaders but also the moderated influence through second- and third-order connections. This results in 200 agents in the network, without exception, leaning towards either positive or negative opinion leaders, underscoring the model’s effectiveness in modeling competitive influence dynamics. The results show that the proposed model enhances the leader’s impact by modeling the influence across a broader network of connections, ensuring that the leader’s opinion is not just accepted but also propagates efficiently through the network.

## 5. Conclusions

This study developed two opinion dynamics models for a single leader subgroup and two leader subgroups with competitive opinions, considering three-stage cascade information transmission and information attenuation. The parameters involved in the models were analyzed through simulation experiments on generated random networks. We found that (1) in the case of a single leader subgroup, the number of leaders had no effect on the follower’s opinion, and when there were two leaders, the followers’ opinions show a biased polarization towards a larger group. (2) When the influence intensity of the target opinion was small, its effect on the opinion leaders and followers was weak. Most followers’ opinions were located between the target opinions. As the influence intensity of the target opinion continued to increase, the leaders’ opinions quickly converged to the target opinion value, so that the followers’ opinions also quickly converged to the target opinion value. (3) The higher the opinion followers’ trust in the leader, the faster the followers’ opinions converged to the leader’s opinion. Conversely, the higher the degree of self-confidence, the easier it was for followers to maintain their original opinion. 

Furthermore, in order to demonstrate the improvements of the proposed method, two comparative experiments were conducted. The first one compared the results of the proposed models on different types and parameters of random networks; the second experiment compared this cascade method with other existing methods. The experimental results indicate the following: (1) The proposed method is able to accelerate the convergence of opinions, which is a critical capability to describe scenarios requiring expedited consensus, such as public emergency responses or rapid information propagation. (2) The model extends its scope to encompass a wider array of network interactions, thereby amplifying the influence of opinion leaders, which ensures the target opinions are effectively disseminated. (3) Although network structure can affect the convergence of opinions, the models reduce the impact of the clustering coefficient and homogeneous coefficient by considering three-level cascading, demonstrating robustness on various types and parameters of random networks, rendering it a versatile instrument for the examination of opinion dynamics across a spectrum of social milieus.

This paper, while offering robust insights, does have its limitations. We acknowledge that the idealized nature of our synthetic networks may not fully encapsulate the intricate and often unpredictable dynamics of real-world social interactions. Moreover, our model’s assumptions about the uniform distribution of initial opinions and the consistent influence of opinion leaders may not hold in reality, where individual predispositions and contextual factors can significantly affect opinion formation and change.

## Figures and Tables

**Table 1:** Distribution of followers’ opinions in Model I with different *N*1 and *N*2.

	N2	10	20	30	40	50
N1						
10		(53%/0%/47%)	(40%/0%/60%)	(34%/1%/65%)	(35%/0%/65%)	(36%/0%/64%)
20		(62%/0%/38%)	(53%/0%/47%)	(45%/0%/55%)	(43%/0%/57%)	(39%/0%/61%)
30		(64%/1%/35%)	(55%/0%/45%)	(53%/0%/47%)	(46%/0%/54%)	(42%/0%/58%)
40		(67%/0%/33%)	(59%/0%/41%)	(54%/0%/46%)	(52%/0%/48%)	(47%/0%/53%)
50		(68%/0%/32%)	(62%/0%/38%)	(57%/1%/42%)	(53%/0%/47%)	(50%/0%/50%)

The three numbers in brackets indicate the distribution of followers with positive (>0.5), neutral ([−0.5, 0.5]), and negative opinions (<−0.5) when *t* = 10.

## Data Availability

The data that support the findings of this study are available by conducting the described simulation experiments with the corresponding settings. The dataset is available on request from the authors.

## References

[1] Mandel A., Venel X. (2020). Dynamic competition over social networks. Eur. J. Oper. Res..

[2] Urena R., Kou G., Dong Y., Chiclana F., Herrera-Viedma E. (2019). A review on trust propagation and opinion dynamics in social networks and group decision making frameworks. Inf. Sci..

[3] Chandrashekaran M., Walker B.A., Ward J.C., Reingen P.H. (1996). Modeling individual preference evolution and choice in a dynamic group setting. J. Mark. Res..

[4] Cheng D., Yuan Y., Wu Y., Hao T., Cheng F. (2022). Maximum satisfaction consensus with budget constraints considering individual tolerance and compromise limit behaviors. Eur. J. Oper. Res..

[5] Liu B., Zhou Q., Ding R.X., Palomares I., Herrera F. (2019). Large-scale group decision making model based on social network analysis: Trust relationship-based conflict detection and elimination. Eur. J. Oper. Res..

[6] Zhou Q., Wu Z., Altalhi A.H., Herrera F. (2020). A two-step communication opinion dynamics model with self-persistence and influence index for social networks based on the degroot model. Inf. Sci..

[7] Bamakan S.M.H., Nurgaliev I., Qu Q. (2019). Opinion leader detection: A methodological review. Expert Syst. Appl..

[8] Zhao Y., Kou G., Peng Y., Chen Y. (2018). Understanding influence power of opinion leaders in e-commerce networks: An opinion dynamics theory perspective. Inf. Sci..

[9] Dong Y., Zhan M., Kou G., Ding Z., Liang H. (2018). A survey on the fusion process in opinion dynamics. Inf. Fusion.

[10] Ding F., Liu Y., Shen B., Si X.M. (2010). An evolutionary game theory model of binary opinion formation. Phys. A Stat. Mech. Its Appl..

[11] Zhan M., Kou G., Dong Y., Chiclana F., Herrera-Viedma E. (2021). Bounded Confidence Evolution of Opinions and Actions in Social Networks. IEEE Trans. Cybern..

[12] Sood V., Redner S. (2005). Voter model on heterogeneous graphs. Phys. Rev. Lett..

[13] Sznajd-Weron K. (2005). Sznajd model and its applications. arXiv.

[14] Chen P., Redner S. (2005). Majority rule dynamics in finite dimensions. Phys. Rev. E—Stat. Nonlinear Soft Matter Phys..

[15] Friedkin N.E., Johnsen E.C. (1990). Social influence and opinions. J. Math. Sociol..

[16] Cao Z., Jiao F., Qu X., Wang W.X., Yang M., Yang X., Zhang B. (2018). Rebels lead to the doctrine of the mean: A heterogeneous degroot model. J. Syst. Sci. Complex..

[17] Deffuant G., Neau D., Amblard F., Weisbuch G. (2000). Mixing beliefs among interacting agents. Adv. Complex Syst..

[18] Hegselmann R., Krause U. (2002). Opinion dynamics and bounded confidence models, analysis, and simulation. J. Artif. Soc. Soc. Simul..

[19] Wu Y., Guo P. (2024). Modeling Misinformation Spread in a Bounded Confidence Model: A Simulation Study. Entropy.

[20] Lorenz J. (2010). Heterogeneous bounds of confidence: Meet, discuss and find consensus!. Complexity.

[21] Jiang L., Liu J., Zhou D., Zhou Q., Yang X., Yu G. (2020). Predicting the Evolution of Hot Topics: A Solution Based on the Online Opinion Dynamics Model in Social Network. IEEE Trans. Syst. Man Cybern. Syst..

[22] Mirtabatabaei A., Bullo F. (2012). Opinion dynamics in heterogeneous networks: Convergence conjectures and theorems. SIAM J. Control Optim..

[23] Dong Q., Zhou X., Martinez L. (2019). A hybrid group decision making framework for achieving agreed solutions based on stable opinions. Inf. Sci..

[24] Dong Y., Ding Z., Martínez L., Herrera F. (2017). Managing consensus based on leadership in opinion dynamics. Inf. Sci..

[25] Li W., Shen H., Huang Z., Yang H. (2023). Research on the Dynamical Behavior of Public Opinion Triggered by Rumor Based on a Nonlinear Oscillator Model. Entropy.

[26] Jia P., MirTabatabaei A., Friedkin N.E., Bullo F. (2015). Opinion dynamics and the evolution of social power in influence networks. SIAM Rev..

[27] Mobilia M. (2003). Does a single zealot affect an infinite group of voters?. Phys. Rev. Lett..

[28] Mobilia M., Petersen A., Redner S. (2007). On the role of zealotry in the voter model. J. Stat. Mech. Theory Exp..

[29] Yildiz E., Ozdaglar A., Acemoglu D., Saberi A., Scaglione A. (2013). Binary opinion dynamics with stubborn agents. ACM Trans. Econ. Comput. (TEAC).

[30] Baumann F., Sokolov I.M., Tyloo M. (2020). A Laplacian approach to stubborn agents and their role in opinion formation on influence networks. Phys. A Stat. Mech. Its Appl..

[31] Ding Z., Chen X., Dong Y., Herrera F. (2019). Consensus reaching in social network DeGroot Model: The roles of the Self-confidence and node degree. Inf. Sci..

[32] Zhao Y., Zhang L., Tang M., Kou G. (2016). Bounded confidence opinion dynamics with opinion leaders and environmental noises. Comput. Oper. Res..

[33] Chen J., Kou G., Wang H., Zhao Y. (2021). Influence identification of opinion leaders in social networks: An agent-based simulation on competing advertisements. Inf. Fusion.

[34] Si X.M., Li C. (2018). Bounded confidence opinion dynamics in virtual networks and real networks. J. Comput..

[35] Glass C.A., Glass D.H. (2020). Social Influence of Competing Groups and Leaders in Opinion Dynamics. Comput. Econ..

[36] Li Q., Du Y., Li Z., Hu J., Hu R., Lv B., Jia P. (2021). HK–SEIR model of public opinion evolution based on communication factors. Eng. Appl. Artif. Intell..

[37] Qin Y., Ma J., Gao S. (2017). Efficient influence maximization under TSCM: A suitable diffusion model in online social networks. Soft Comput..

[38] Min B., San Miguel M. (2023). Threshold cascade dynamics in coevolving networks. Entropy.

[39] Kang M., Liang T., Sun B., Mao H.Y. (2023). Detection of opinion leaders: Static vs. dynamic evaluation in online learning communities. Heliyon.

[40] Winter S., Neubaum G. (2016). Examining characteristics of opinion leaders in social media: A motivational approach. Soc. Media + Soc..

[41] Turnbull P.W., Meenaghan A. (1980). Diffusion of innovation and opinion leadership. Eur. J. Mark..

[42] Flynn L.R., Goldsmith R.E., Eastman J.K. (1996). Opinion leaders and opinion seekers: Two new measurement scales. J. Acad. Mark. Sci..

[43] Pan L., Shao H., Li D. (2022). Peer selection in opinion dynamics on signed social networks with stubborn individuals. Neurocomputing.

[44] Van Eck P.S., Jager W., Leeflang P.S. (2011). Opinion leaders’ role in innovation diffusion: A simulation study. J. Prod. Innov. Manag..

[45] Das A., Gollapudi S., Munagala K. Modeling opinion dynamics in social networks. Proceedings of the 7th ACM International Conference on Web Search and Data Mining.

[46] Couzin I.D., Krause J., Franks N.R., Levin S.A. (2005). Effective leadership and decision-making in animal groups on the move. Nature.

[47] Hu B., Hu X. (2018). Qualitative modeling of catastrophe in group opinion. Soft Comput..

[48] Gargiulo F., Gandica Y. (2017). The role of homophily in the emergence of opinion controversies. J. Artif. Soc. Soc. Simul..

[49] Lima F.W., Sousa A.O., Sumuor M.A. (2008). Majority-vote on directed Erdős–Rényi random graphs. Phys. A Stat. Mech. Its Appl..

[50] Albert R., Barabási A.L. (2002). Statistical mechanics of complex networks. Rev. Mod. Phys..

